# Effective Saccharification of Corn Stover Using Low-Liquid Aqueous Ammonia Pretreatment and Enzymatic Hydrolysis

**DOI:** 10.3390/molecules23051050

**Published:** 2018-05-01

**Authors:** Nguyen Phuong Vi Truong, Tae Hyun Kim

**Affiliations:** 1 Faculty of Natural Sciences, Thu Dau Mot University, Thu Dau Mot City 820000, Binh Duong Province, Vietnam; vitnp@tdmu.edu.vn; 2 Department of Materials Science and Chemical Engineering, Hanyang University, Ansan, Gyeonggi-do 15588, Korea

**Keywords:** aqueous ammonia, alkaline pretreatment, enzymatic digestibility, lignocellulosic biomass, cellulosic sugar

## Abstract

Low-liquid aqueous ammonia (LLAA) pretreatment using aqueous ammonia was investigated to enhance enzymatic saccharification of corn stover. In this method, ground corn stover was simply contacted with aqueous ammonia mist (ammoniation step), followed by pretreatment at elevated temperature (90–150 °C) for an extended period (24–120 h) at different solid/liquid (S/L) ratios (0.29, 0.47 or 0.67), termed a pretreatment step. After that, excess (unreacted) ammonia was removed by evaporation, and the pretreated material was immediately saccharified by an enzyme without a washing step. The effects of key reaction parameters on both glucan digestibility and XMG digestibility were evaluated by analysis of variance (ANOVA). Under the best pretreatment conditions [S/L = 0.47, 0.16 (g NH_3_)/(g biomass), 90 °C, 24 h], LLAA pretreatment enhanced enzymatic digestibility from 23.1% for glucan and 11.3% for XMG (xylan + galactan + mannan) of untreated corn stover to 91.8% for glucan and 72.6% for XMG in pretreated solid.

## 1. Introduction

Limited supplies of fossil resources, climate change due to carbon dioxide accumulation in the atmosphere, and increased demand for fuels and chemicals have triggered an increase in utilization of diverse renewable feedstock. To implement the production of a wide range of fuels, chemicals, and materials from renewable sources, most green research in recent years has focused on the development of renewable fuels and bio-based chemicals as a substitute for conventional fossil fuels (gasoline and diesel) and petroleum-based chemicals. In particular, cellulosic fuel ethanol, a second-generation biofuel, has the potential to solve several problems, including limited feedstock availability and food competition with fuel, that are currently associated with first-generation biofuels such as fuel ethanol from corn starch or sugarcane [1]. Cellulosic ethanol can be produced from inexpensive and abundant lignocellulosic materials such as woody biomass and herbaceous biomass [2]. Therefore, it is currently believed that cellulosic ethanol can meet a larger proportion of global transportation fuel demand in the near future. Production of ethanol from lignocellulosic biomass is still challenging because of the recalcitrant nature of the latter; for example, lignin is an inhibitor of enzymatic and microbial reactions and has high crystallinity and complex chemical composition [3,4]. Unlike sugar and starch, carbohydrates of lignocellulosic biomass consist of five different sugar units (glucose, xylose, arabinose, galactose, and mannose). To utilize lignocellulosic biomass effectively, production of fuels and chemicals from all sugars is necessary [5].

Currently, most of fuel ethanol is being produced from corn starch or sugarcane in many countries, such as China, Brazil, and the United States. Corn stover includes husk, leaves, and stalk that are left in the field after grain harvest and is a co-product of corn grain production. Therefore, manufacture of fuel ethanol from corn stover may be a reasonable approach to commercialization of the first cellulosic ethanol process at present [1].

Because of the aforementioned difficulties with utilization of lignocellulosic biomass, pretreatment is necessary to disrupt the recalcitrant structure of the plant cell walls, thus enabling easy access to production of fermentable sugar, which is then fermented to produce ethanol [6]. Therefore, study in recent years has been focused on the development of effective pretreatment method intended to make the lignocellulosic sugars available for ethanol conversion. Nonetheless, it is known that most of pretreatment methods involving various acids and alkalis at high temperature typically generate inhibitory products such as phenolic compounds, furfural, 5-hydroxymethylfurfural, and aldehydes. Therefore, some alkaline pretreatments under mild reaction conditions are considered viable pretreatment methods for different types of lignocellulosic biomass such as wood biomass and herbaceous biomass with high lignin content [7]. In a large-scale biomass conversion process involving a pretreatment unit, the chemical and water inputs can be a critical factor for the development of a commercially viable biochemical method. Nevertheless, a washing step is typically required in both acid and alkali pretreatment methods for the removal of the remaining chemical reagents from the chemically treated biomass, and the recovery and reuse of water and chemicals significantly affect the total energy cost of the biomass conversion process.

To reduce the water and chemical inputs into biomass processing, our laboratory previously reported that a pretreatment method using anhydrous ammonia (low-moisture anhydrous ammonia; LMAA) effectively improves the enzyme saccharification yield of agricultural biomass [8,9,10]. Although the LMAA method has been developed to eliminate the washing step, one of the drawbacks of anhydrous (gaseous) ammonia is that it must be stored and handled under high pressure, which requires specially designed and well-maintained high-pressure equipment and systems during biomass processing.

**Figure 1:**
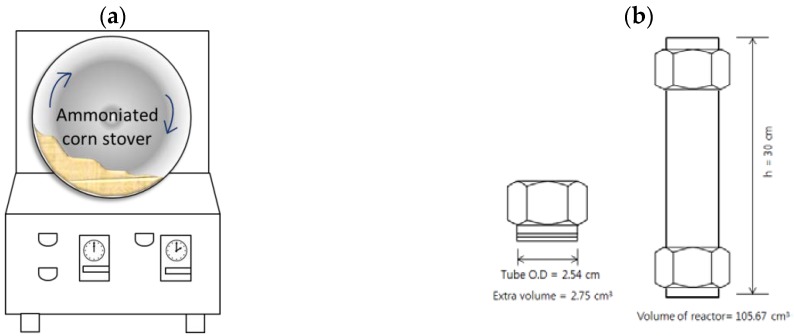
Tumbler mixer (**a**) and batch-type pretreatment reactor (**b**).

In our present study, low-liquid aqueous ammonia (LLAA) pretreatment was proposed to solve such problems associated with a process using gaseous ammonia. This pretreatment method consists of ammoniation, pretreatment, and evaporation steps; i.e., corn stover is well contacted with aqueous ammonia mist using nozzle spray and tumbler mixer (Figure 1a) (ammoniation step), followed by pretreatment step at an elevated temperature (up to 150 °C) for an extended period (up to 120 h) using a tight-sealed batch reactor (Figure 1b). After that, excess (unreacted) ammonia is removed by evaporation, and the resulting material can be immediately saccharified by a commercial cellulase without a washing step. LLAA pretreatment can be expected to lower the operating cost because it requires low input of liquid (reagents and water). Furthermore, aqueous ammonia is easy to handle, making this method a more industrially adoptable process for an upcoming biomass-processing facility.

## 2. Results and Discussion

### 2.1. Effects of Reaction Temperature and Time on the Chemical Composition of Pretreated Corn Stover

The initial composition of the untreated corn stover is summarized in Table 1.

**Figure 2:**
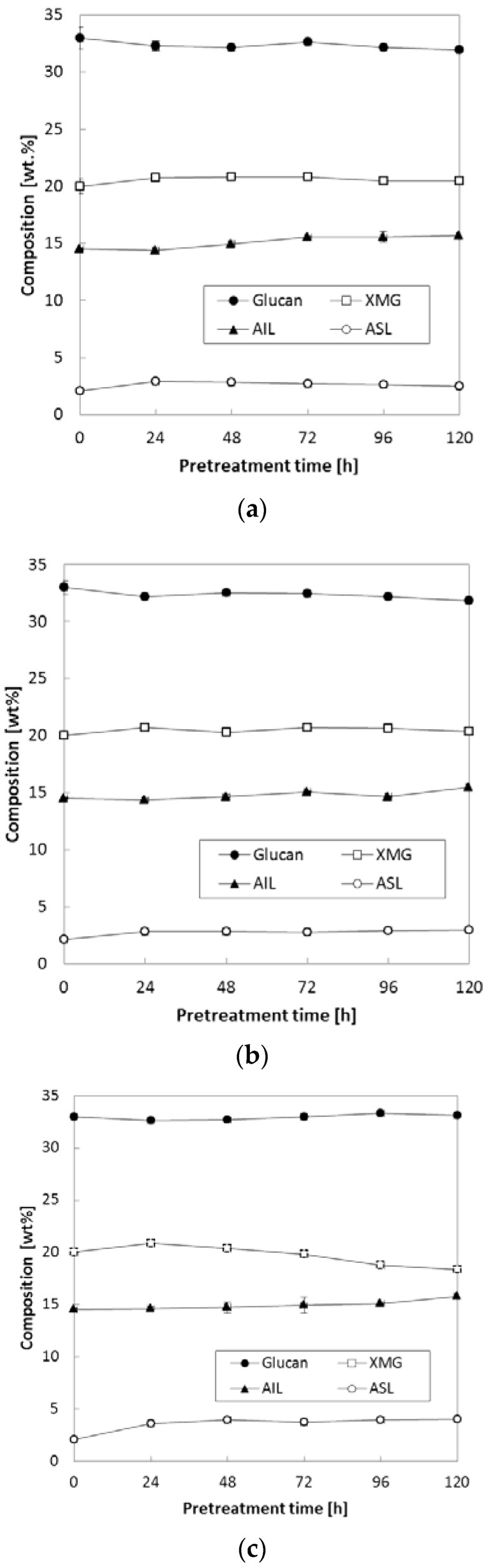
Effects of pretreatment temperature and time on the composition of pretreated corn stover. Pretreatment: 0.16 (g NH_3_)/(g biomass), S/L ratio = 0.47, 24–120 h. (**a**) 90 °C, (**b**) 120 °C, (**c**) 150 °C. The data in the figure show mean values.

The effects of reaction temperature and time were evaluated, and Figure 2 presents the changes in chemical composition at various pretreatment temperatures with extended pretreatment periods. Three pretreatment temperatures (90, 120 and 150 °C) were applied during extended pretreatment periods (24–120 h) while we kept other conditions constant [0.16 (g NH_3_)/(g biomass), S/L = 0.47]. As shown in Figure 2a,b, pretreatment at lower temperatures (90 and 120 °C) did not result in significant changes in carbohydrates (glucan and XMG) and lignin (acid-insoluble lignin, AIL and acid-soluble lignin, ASL) even with a prolonged reaction period (up to 120 h). On the other hand, there was a marginal change in both XMG and lignin contents at 150 °C (Figure 2c), in particular, after 72–96 h of pretreatment. Pretreatment at a high temperature (150 °C) for 120 h increased both AIL and ASL contents to 15.8% and 4.1%, respectively, which represented 1.3% and 2.0% increases as compared to untreated corn stover (Figure 2c). On the contrary, XMG content decreased from 20.0% of untreated corn stover to 18.4% after pretreatment at 150 °C for 120 h. Glucan content was maintained well at all three temperatures of pretreatment.

### 2.2. The Effect of the S/L Ratio on Chemical Composition of Pretreated Corn Stover

**Figure 3:**
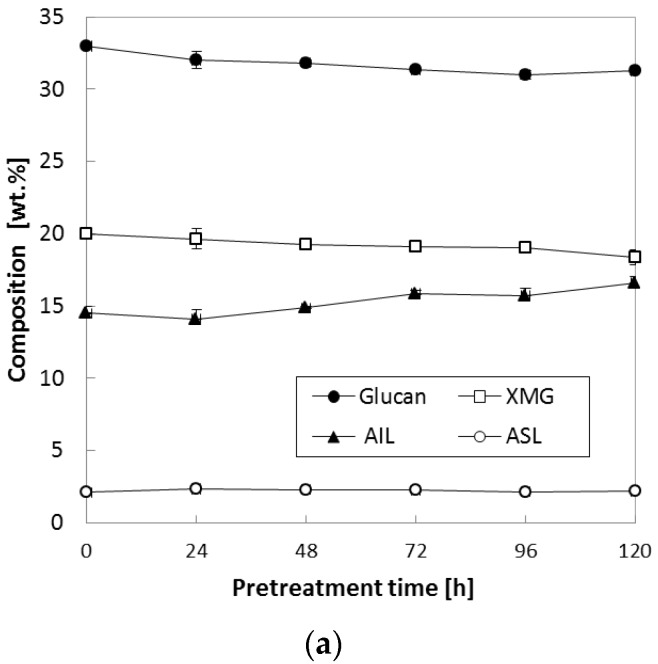

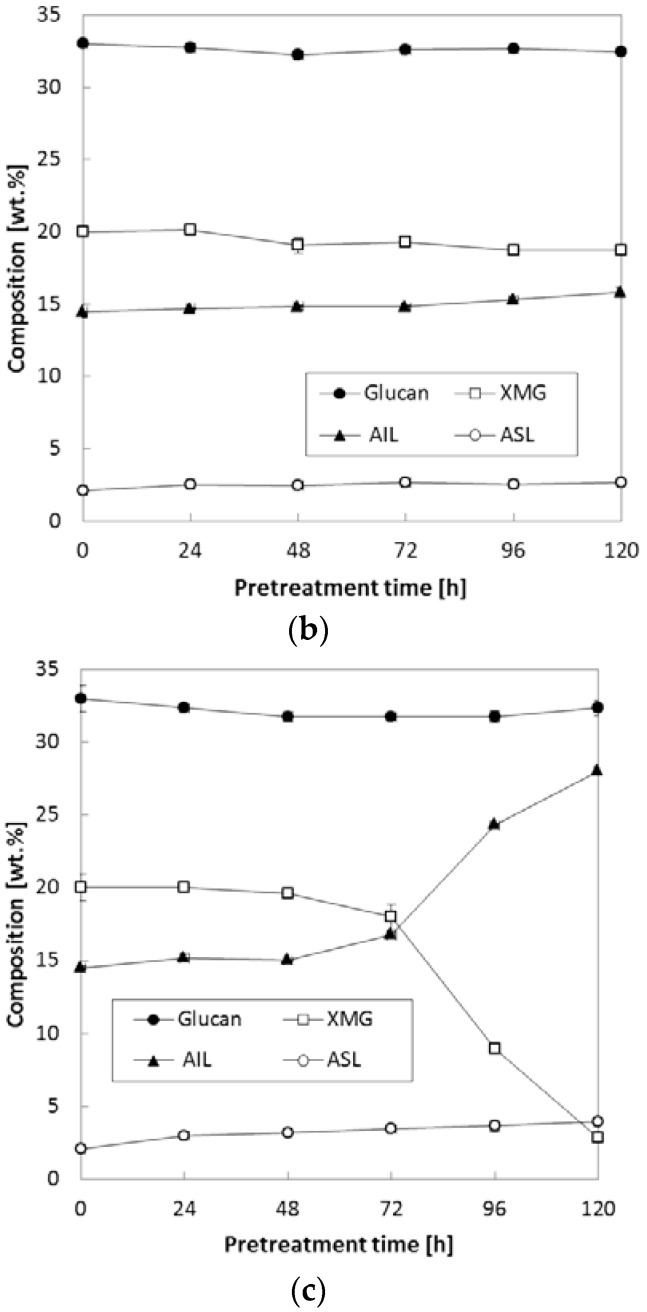
Effects of the S/L ratio on composition of corn stover. Pretreatment: 150 °C, 24–120 h, 0.08 (g NH_3_)/(g biomass) (**a**) S/L ratio = 0.29, (**b**) S/L ratio = 0.47, (**c**) S/L ratio = 0.67. The data in the figure show mean values.

In the above test, various temperatures (90–150 °C) were tested while we kept NH_3_ loading at 0.16 (g NH_3_)/(g biomass) and S/L ratio at 0.47. Because it was found that chemical composition was more affected at 150 °C than other temperature (90 °C and 120 °C), another set of experiments to study the compositional changes during pretreatment was conducted at low NH_3_ loading [0.08 (g NH_3_)/(g biomass)] at various S/L ratios. When the S/L ratio was varied between 0.29 and 0.47, both XMG and lignin were slightly affected; i.e., as the reaction time increased, XMG content gradually decreased from 20.0% of untreated corn stover to 18.7–19.0% of pretreated corn stover, while both AIL and ASL increased accordingly; in particular, AIL increased from 14.5% of untreated corn stover to 15.8–16.6% of pretreated corn stover (Figure 3a,b). Nevertheless, it was found that the increase in the S/L ratio did not result in a considerable change in glucan content under all the tested conditions. Most significant changes in XMG and AIL occurred in case of the pretreatment at the highest S/L ratio (S/L = 0.67) and reaction time >72 h (Figure 3c). XMG content decreased from 20.0% of untreated corn stover to 2.9% of 120-h pretreated corn stover, whereas AIL increased from 14.5 to 28.0%. ASL content was slightly increased by ammonia pretreatment (from 2.1% of untreated corn stover to 4.0% of pretreated corn stover) as reaction time was extended to 120 h. The reason for the lignin upregulation during pretreatment under the harsh conditions (Figure 3c) was not clear at this stage. This observation was consistent with our previous report about the pretreatment of herbaceous biomass using gaseous ammonia; i.e., pretreated corn stover at 130–150 °C showed a considerable change in the composition of treated solids [8]. Nevertheless, it could be hypothesized according to the literature that the pretreatment reaction in the presence of water and the chemical depolymerize the linkages in the lignin–carbohydrate complex; this action results in removal of lignin along with other fiber fragments from cellulose and hemicellulose and, if they are not removed promptly, causes its subsequent repolymerization [11]. XMG is the main component of hemicellulose in herbaceous plants [12] and can easily be degraded during chemical pretreatment at a high temperature with a long reaction period [13,14]. This repolymerized lignin contains residual xylan and other degradation products becoming acid-insoluble complexes that are not hydrolyzed by sulfuric acid during chemical composition analysis following standard laboratory analytical procedure (LAP) of the National Renewable Energy Laboratory (NREL; Golden, CO, USA), thus resulting in increased measured lignin amounts [15,16,17,18]. In addition, another study indicates that the degraded hemicellulose/cellulose forms pseudo-lignin [19], which can affect lignin analysis.

### 2.3. The Effect of NH_3_ Loading on Enzymatic Digestibility of Pretreated Solids

In the above test (Section 2.2), high S/L ratio (0.67) resulted in significant decomposition of sugar, in particular, XMG during pretreatment, which was not desirable feature for an effective pretreatment for high sugar conversion yield [20,21]. To evaluate the effect of NH_3_ loading on enzymatic digestibility, three different NH_3_ loads [0.08, 0.16, or 0.24 (g NH_3_)/(g biomass)] were applied while other conditions were kept constant (S/L = 0.47, reaction temperature 90 °C, and reaction time 24 h), and Table 2 summarizes the chemical composition data and enzymatic digestibility (at 72 h of the hydrolysis reaction) of the pretreated corn stover. An interesting trend was observed with increased NH_3_ loading: glucan digestibility of the pretreated solid sample increased from 71.6 to 91.8% with NH_3_ loading up to 0.16 (g NH_3_)/(g biomass), then decreased to 84.7% at 0.24 (g NH_3_)/(g biomass) loading. The XMG digestibility showed a similar trend: it increased from 66.7 to 72.6% when NH_3_ loading was increased from 0.08 to 0.16 (g NH_3_)/(g biomass) and decreased again above that NH_3_ loading [66.5% at 0.24 (g NH_3_)/(g biomass)]. Although it was unclear in the present step, it was assumed that a change in chemical composition may play a role in enzymatic saccharification.

**Figure 4:**
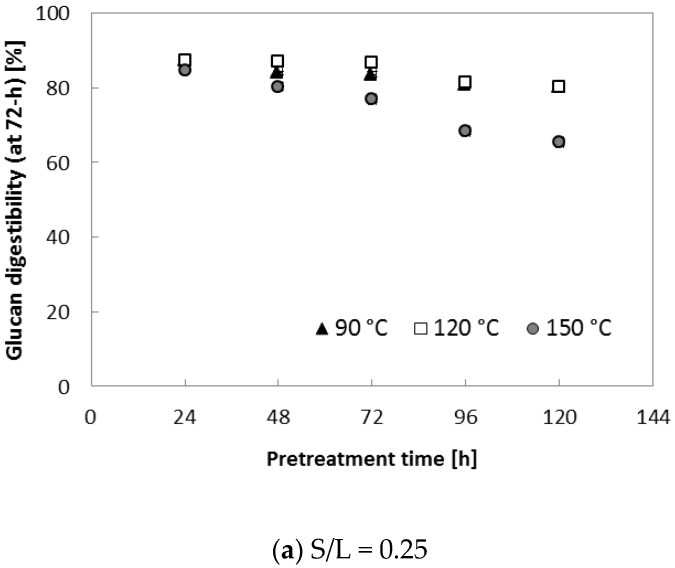

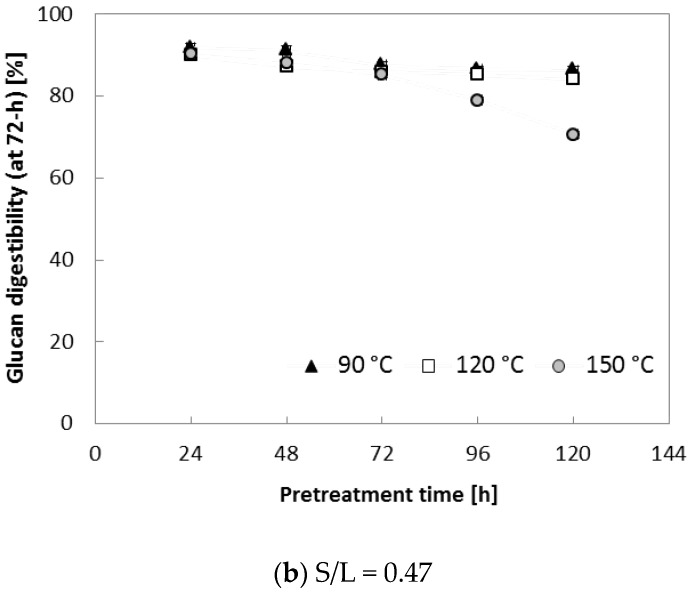
Glucan digestibility at elevated pretreatment temperature. Pretreatment: 0.16 (g NH_3_)/(g biomass), 90–150 °C, 24–120 h, (**a**) S/L ratio = 0.29, (**b**) S/L ratio = 0.47. Enzymatic hydrolysis conditions: 15 (FPU CTec2)/(g glucan) loading, 50 °C, 150 rpm, hydrolysis time: 72 h. The data in the figure show mean values (standard deviation < 1.5).

To further evaluate the effect of various S/L ratios on enzymatic saccharification, two different S/L ratios (0.29 and 0.47) were applied. Ammonia loading of 0.16 (g NH_3_)/(g biomass) was used because it resulted in the highest digestibility (91.8% for glucan and 72.6% for xylan in Table 2). In this set of tests, three temperatures (90, 120 and 150 °C) with increased pretreatment time (~120 h) were applied to each S/L ratio (0.29 and 0.47). Figure 4 indicates that pretreatment at 150 °C for an extended treatment period (>72 h) resulted in lower glucan digestibility (71–85% at S/L = 0.29, 65–72% at S/L = 0.47) in comparison with the samples treated for 24–48 h (88–90% at S/L = 0.29, 82–84% at S/L = 0.47). It was assumed that higher lignin content (AIL) of pretreated corn stover at the high temperature (150 °C) contributed to the reduced enzymatic digestibility, in agreement with results from another study [22]. Owing to the improved enzymatic digestibility (Figure 4), 90 °C and 24 h were selected as the best pretreatment conditions for a further experiment (described in the following section); these conditions were assumed to be desirable because milder reaction conditions (90 °C and 24 h) are preferred for a reduction in the operating cost in a large-scale biomass conversion process.

### 2.4. Analysis of Variance (ANOVA)

To assess possible correlations of the effects between various reaction parameters and enzymatic digestibility, the single and combined effects of various factors on both glucan digestibility and XMG digestibility were evaluated by ANOVA, and the performance data are shown in Table 3. Among various reaction conditions, only the combined coefficient of “Temp × Time” had a *p* value less than 0.05 (*p* = 0.0233 for glucan digestibility and *p* = 0.0370 for XMG digestibility), implying that this coefficient significantly affects both glucan and XMG digestibility levels simultaneously, while other coefficients did not have a significant effect on enzymatic digestibility or influenced on either glucan or XMG digestibility. Therefore, the pretreatment temperature–time may be considered primary factors that can enhance the pretreatment effectiveness. In addition, the reaction temperature (Temp) seemed to have a significant effect on glucan digestibility (*p* = 0.0182) and showed a clear-cut tendency (close to significance) to affect XMG digestibility (*p* = 0.0511). On the other hand, the coefficient of time (reaction time), NH_3_ (ammonia loading), and S/L and combined coefficient of “Time × S/L” and “S/L × NH_3_” had lower influence on both glucan and XMG digestibility (*p* > 0.05). The combined coefficient of “Temp × S/L” had a *p* value less than 0.05, indicating that this coefficient significantly affects the glucan digestibility, whereas the combined coefficient of “Temp × NH_3_” and “Time × NH_3_” had a *p* value less than 0.05, suggesting that there is a significant effect on XMG digestibility.

As discussed previously, the alkaline treatment such as the use of an ammonia solution can remove lignin and thereby increase the digestibility of biomass [18,23,24]. It was assumed that increasing the ammonia loading caused the breakdown of ester bonds in hemicellulose and lignin polymers at the elevated temperature; this situation consequently can improve the enzymatic hydrolysis of hemicellulose (XMG).

### 2.5. Residual Ammonia

Although ammonia can be evaporated and removed due to its high volatility, some of the impregnated ammonia cannot be easily removed and was assumed to affect the saccharification of fibers during enzymatic hydrolysis. The effect of residual ammonia content on enzymatic digestibility was evaluated, but it was assumed that residual ammonia content does not solely affect enzymatic digestibility because the level of residual ammonia content can be strongly influenced by other reaction parameters such as ammonia loading, pretreatment temperature, pretreatment time, the S/L ratio, and the combined effects of these parameters.

An evaluation assay of the effect of residual ammonia content on glucan digestibility was conducted for each reaction parameter. The effect of reaction severity on residual ammonia content was evaluated under various reaction conditions and the *R*^2^ values as the predicted probability are summarized in Table 4. Because four different reaction parameters were compared, we categorized each different reaction condition into three different severity levels such as low, medium, and high severities. The higher severity means severe treatment conditions (see the note in Table 4). The *R*^2^ values in Table 4 indicate that samples treated at S/L ratios corresponding to low and high severity resulted in a relatively strong correlation between residual ammonia content and glucan digestibility (*R*^2^ = 0.3950 and 0.5607, respectively). In addition, samples treated with ammonia loading of medium severity showed *R*^2^ = 0.4113, which indicated some correlation between residual ammonia content and glucan digestibility. Overall, the coefficients (*R*^2^) of the trend lines were 0.027–0.5607; therefore, the model equations of the trend lines were not significant.

**Figure 5:**
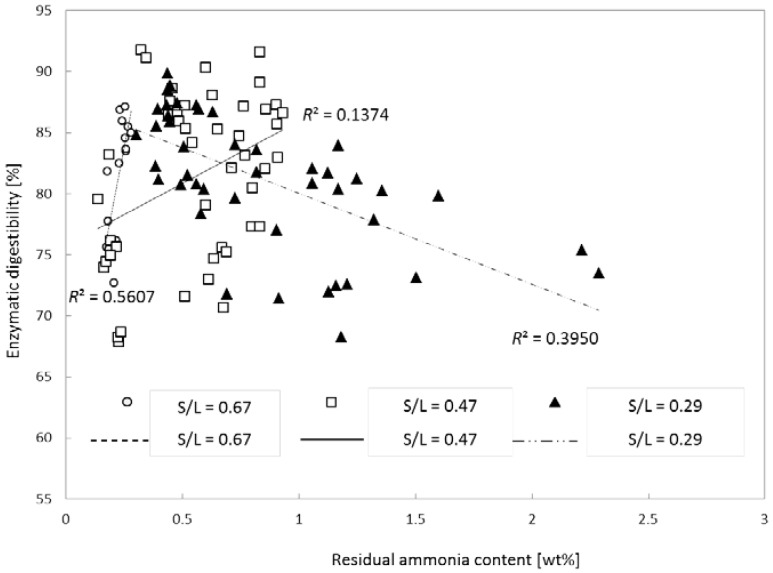
Effects of residual ammonia content on glucan digestibility of pretreated corn stover. Pretreatment: 0.08, 0.16 or 0.24 (g NH_3_)/(g biomass), S/L ratio = 0.29, 0.47 or 0.67, 24–120 h, 90–150 °C. Enzymatic hydrolysis: 15 (FPU CTec2)/(g glucan) loading, 50 °C, 150 rpm, 72 h.

Because it was found that the S/L ratio had the strongest effect (Figure 3 and Figure 4), we next evaluated the effect of increasing S/L. Besides, residual ammonia was significantly affected (*p* < 0.05; data not shown) when S/L was changed. Figure 5 presents the relation of enzymatic digestibility (at 72 h of hydrolysis) of glucan with residual ammonia content at different S/L ratios. The *R*^2^ values of three different S/L levels indicated that there was no clear trend between glucan digestibility and residual ammonia content even though the treated samples with high concentrations of residual ammonia seemed to have slightly lower digestibility than did the samples with low residual ammonia content. It should also be noted that the residual ammonia in the pretreated biomass can serve as an essential nitrogen source for microbial cell growth during fermentation if it is at an appropriate concentration.

### 2.6. Mass Balance

**Figure 6:**
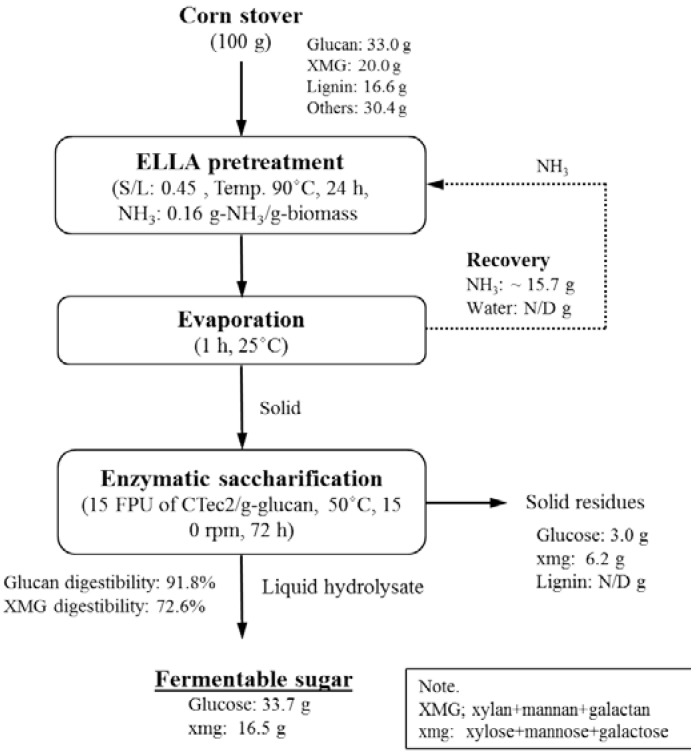
Schematic diagram and mass balance during conversion of corn stover to sugars.

Figure 6 summarizes the overall mass balance for the process of conversion of 100 g of corn stover to fermentable sugar by pretreatment under the best conditions [S/L = 0.47, 0.16 (g NH_3_)/(g biomass), 90 °C, 24 h]. One of the features of LLAA method is that it does not solubilize any component during pretreatment and only modifies lignin and hemicellulose. Therefore, the input and output of the whole sugar conversion process are almost the same. The residual ammonia and ammonia recovery in this calculation were 1.7 wt % residual ammonia [0.16 (g NH_3_)/(g biomass)] and 98.3% (15.7 g) after pretreatment and evaporation, respectively. Next, the pretreated solids were saccharified by means of 15 (FPU (filter paper unit) CTec2)/(g glucan) at 50 °C, 150 rpm, 72 h, and 1.0% (*w*/*v*) glucan loading. The highest glucan and XMG digestibility at an enzyme load of 15 FPU/(g glucan) was 91.8% and 72.6%, respectively. According to the mass balance in Figure 6, 33.7 g of glucose and 16.5 g of xmg were produced from 100 g of corn stover. The residue after enzymatic saccharification mostly consisted of lignin, ash, and unconverted polysaccharides.

### 2.7. Comparison of Various Ammonia Pretreatments

Alkaline pretreatment is considered an effective way to break down the structure of lignin and therefore to enhance the enzymatic hydrolysis of lignocellulosic biomass [25]. Table 5 shows a comparison of the features and reaction conditions of various alkaline pretreatment methods (in particular, methods involving ammonia). Pretreatment methods shown in Table 5 include low-liquid ammonia recycle percolation (LLARP), soaking in aqueous ammonia (SAA), LMAA, and LLAA [8,10,23,26,27].

Among the methods listed in Table 5, LMAA requires the least amount of chemical loading [0.1 (g NH_3_)/(g biomass)], and LLAA is the next best method [0.16 (g NH_3_)/(g biomass)] and shows the same water consumption [<1.0 (g H_2_O)/(g biomass)]. The sugar production process using these two pretreatment methods can be considered more economical than those based on other pretreatment methods [0.5–0.9 (g catalyst)/(g biomass) and 2.8–10 (g H_2_O)/(g biomass)]. Furthermore, in contrast to other methods (LLARP and SAA) in Table 5, the most desirable feature of LLAA and LMAA is that the washing step after pretreatment is not necessary; this feature can reduce the water consumption and thus reduce total energy cost in the biomass conversion process. In terms of severity of pretreatment conditions, LLAA, LMAA, and SAA processes involve mild reaction conditions. Although LLARP requires a short reaction period (~10 min), it should be carried out at high temperature (170 °C), while the other three ammonia pretreatment methods (LLAA, LMAA, and SAA) require more time (12–24 h) at a moderate temperature (60–90 °C). On the other hand, the longer pretreatment time and large water input in the SAA method are required even though it involves a mild reaction temperature; these characteristics are not considered desirable for an economically viable process [28].

## 3. Materials and Methods

### 3.1. Materials

#### 3.1.1. Feedstock

Corn was grown and harvested in China in September 2015, and corn stover was then collected and provided by CJ Cheiljedang Co. (Seoul, Korea). The received corn stover was air-dried at ambient temperature (~25 °C), ground up, passed through a sieve with a mesh size of 10–35 mesh (US Standard, 0.5–2.0 mm of nominal sieve opening) sieves, and then stored in sealed plastic containers at ambient temperature. The initial composition of the biomass was determined by a standard LAP of the NREL (Table 1) [29]. It should be noted that glucan, xylan, and lignin are the main components among the various ones shown in Table 1; therefore, an evaluation of pretreatment effects was focused on those three components in this study.

Ammonium hydroxide (28.0–30.0%; lot number A29260I1) and sulfuric acid (ACS grade, 95–98%, lot number SZBF0140V) were purchased from Daejung Chemical & Metals Co., Ltd. (Shehung-si, Gyeonggi-do, Korea) and Sigma-Aldrich (St. Louis, MO, USA), respectively. Avicel^®^ PH-101 (catalog number 900-3-6, lot number BCBJ029V, Sigma-Aldrich) was acquired and served as a control sample in the enzymatic-digestibility test.

#### 3.1.2. Enzymes

Cellic^®^ CTec2 (batch number: VCP10006, Novozymes Inc., Bagsvaerd, Denmark) was used for enzymatic saccharification of untreated and pretreated corn stover. The average activity of the enzyme, as determined by the LAP of the NREL was 88.91 FPU/mL [30].

### 3.2. Pretreatment

#### 3.2.1. The First Step: Ammoniation

To apply ammonia loading at different target concentrations [0.08, 0.16 or 0.24 (g NH_3_)/(g biomass)], an ammonium hydroxide (NH_4_OH) solution at various solid/liquid (S/L) ratios (0.29, 0.47 or 0.67) was added in the form of mist using nozzle spray and tumbler mixer. The S/L ratio was calculated as follows:

$$S/L=\frac{\mathrm{Total}\;\mathrm{solids}\;\left(g\right)}{\mathrm{Total}\;\mathrm{solids}\;\left(g\right)+\mathrm{water}\;\&\;\mathrm{moisture}\;\left(g\right)}.$$

The initial moisture content of corn stover was approximately 8.5% and was loaded for ammoniation. After spraying of ammonium hydroxide mist, corn stover (100 g, dry basis) was homogenized at 30 rpm for 1 h in the tumbler mixer shown in Figure 1a.

#### 3.2.2. The Second Step: Pretreatment

Ammoniated corn stover treated with aqueous ammonia (10 g, dry basis) was packed in a smaller sealed batch reactor (30.0-cm length, 2.54-cm internal diameter [ID], and 0.21-cm tube wall thickness [internal volume: 105.7 mL]; Figure 1b). Openings of the sealed batch reactor were tightened carefully enough to prevent ammonia leaking. The reactor was placed in the forced convection oven (model no. OF-22GW, Jeio Tech Co., Ltd., Daejeon, Korea) and then heated from ambient temperature to the target temperatures (90–150 °C) in 1 h and maintained at the desired temperature for 24–120 h.

#### 3.2.3. The Third Step: Evaporation

After completion of the pretreatment process, the reactors were cooled down to ambient temperature. The reactors were then opened, and the treated sample was transferred into a tray. The collected sample was placed in the fume hood to remove excess ammonia by evaporation for 1 h at 25 °C. One portion of the sample was used for analysis of residual ammonia content, and the other portion was used for composition analysis.

### 3.3. Analytical Methods

Soxhlet extraction was applied to determine the water- and ethanol-soluble extractives of untreated corn stover. A two-step Soxhlet extraction was conducted; the first step of extraction with de-ionized (DI) water for 8 h was followed by the second step of extraction with ethanol (190 proof) for 24 h.

The chemical composition of untreated and pretreated corn stover was analyzed for carbohydrates, AIL, ASL (on a UV spectrophotometer at 320 nm), and ash (a gravimetric method involving a muffle furnace at 575 °C) following the NREL LAP [29]. Carbohydrate contents were determined by means of a high-performance liquid chromatography (HPLC) system (Shimadzu LC-10A, Shimadzu Inc., Kyoto, Japan) equipped with Bio-Rad Aminex HPX-87P (catalog number 1250098; Bio-Rad Inc., Hercules, CA, USA) and an 87H column (catalog number 1260140; Bio-Rad Inc., Hercules, CA, USA) and a refractive index detector (model RID-10A, Shimadzu Inc., Kyoto, Japan). Analytical conditions for HPLC were as follows: mobile phase of water (0.6 mL/min) at column temperature of 85 °C and 0.005 M H_2_SO_4_ (0.6 mL/min) at 65 °C for the HPX-87P column and HPX-87H column, respectively.

### 3.4. Enzymatic Digestibility

This property of pretreated and untreated corn stover was evaluated in duplicate in rubber-capped 250-mL Erlenmeyer flasks containing 100 mL of a liquid and 1.0 g of a glucan loading (3.0 g of pretreated solid loading, dry basis) according to the NREL-LAP [30]. The recovered solid samples obtained after the evaporation were used directly in the enzymatic digestibility tests without drying. Reaction conditions for the digestibility test were 50 °C, pH 4.8, and 150 rpm at 15 FPU/(g glucan) enzyme load in 0.05 M citrate buffer. Each sample in 100-mL working volume was saccharified in a shaking incubator (model number VS 8480SFN, Vision Scientific Co., Ltd., Daejeon, Korea). Total glucose content after 72 h of hydrolysis was used to calculate the enzymatic digestibility. Avicel^®^ PH-101 was also put through the same digestibility test conditions and served as a control sample. The glucan and XMG digestibility values were calculated as follows:

$$\mathrm{Glucan}\;\mathrm{digestibility}=\frac{\mathrm{Total}\;\mathrm{released}\;\mathrm{glucose}\;\left(g\right)\;\times \;0.9}{\mathrm{Initial}\;\mathrm{glucan}\;\mathrm{loading}\;\left(g\right)}\;\times \;100,$$

where 0.9 is the factor for conversion of glucose to equivalents of glucan.

$$\mathrm{XMG}\;\mathrm{digestibility}=\frac{\mathrm{Total}\;\mathrm{released}\;\mathrm{XMG}\;\left(g\right)\;\times \;0.88}{\mathrm{Initial}\;\mathrm{XMG}\;\mathrm{loading}\;\left(g\right)}\;\times \;100,$$

where 0.88 is the factor for conversion of xylose to equivalents of XMG.

### 3.5. Residual Ammonia Analysis

One gram of untreated and pretreated samples was placed in a glass bottle with 80 mL of a 1.0% borate buffer solution. These glass bottles were placed in a convection oven at a stable temperature (80 °C) and incubated there for 24 h. After that, the glass bottles with residual ammonia in the liquid were removed from the oven. Liquid and solids were separated by filtration through filter paper (Fisher catalog number F2044-090, size: 90 mm Ø, pack: 100 units from CHmlab Group, Barcelona, Spain). Then, the filtrate was diluted to 100-mL working volume. The liquid, which contained ammonia, was reacted with a 10 N sodium hydroxide (NaOH) solution. Residual ammonia content in the liquid was determined by means of an ammonia analyzer (model Accumet^®^, XL250, Dual Channel pH/mV/Ion, Thermo Fisher Scientific Inc., Tampa, FL, USA) and an ion-selective electrode (ISE, Fisher catalog number 13-620-509).

### 3.6. ANOVA

The statistical analysis of the data was performed using SAS^®^ software (version 9.4, SAS Institute Inc., Cary, NC, USA).

## 4. Conclusions

LLAA pretreatment can reduce energy use because it requires lesser inputs of ammonia and water as compared to other pretreatment technologies, and can enable economically viable processes. In addition, the LLAA pretreatment has advantages over previously developed ammonia pretreatment methods, e.g., it uses aqueous ammonia without washing. Therefore, this approach can be regarded as a more economically feasible technology for scaling up. Moreover, LLAA shows promise because of the effectiveness of this pretreatment at enhancing enzymatic digestibility of corn stover. The highest glucan and XMG digestibility levels were 91.8% and 72.6%, respectively, at 15 FPU/(g glucan) enzyme loading.

## Figures and Tables

**Table 1:** Chemical composition of untreated corn stover.

Sample ID	Corn Stover
	[wt %]
Extractive	
Glucose	0.1 ± 0.2
Sucrose	1.1 ± 0.0
Fructose	1.2 ± 0.0
Soluble lignin	1.3 ± 0.0
Other extractives	16.5 ± 0.0
Extractives-free solid	
Glucan	33.0 ± 0.6
Xylan	17.9 ± 0.1
Arabinan	3.2 ± 0.5
Mannan	0.2 ± 0.0
Galactan	1.9 ± 0.2
AIL ^1^	14.5 ± 0.1
ASL ^2^	2.1 ± 0.0
Ash	0.9 ± 0.1
Protein	6.0 ± 0.0
Total	100

Note: All weight percentages were calculated on the basis of oven-dried biomass weight; ^1^ AIL: acid-insoluble lignin; ^2^ ASL: acid-soluble lignin SD: standard deviation (*n* = 3).

**Table 2:** Effects of ammonia loading on composition and enzymatic digestibility.

NH_3_ Loading	Composition			Enzymatic Digestibility (at 72 h)	
	Glucan	XMG	Lignin	Glucan	XMG
(g NH_3_)/(g Biomass)	(wt %)	(wt %)	(wt %)	(%)	(%)
Untreated	33.0 ± 0.8	20.0 ± 0.4	16.6 ± 0.9	23.0 ± 2.1	11.3 ± 1.2
0.08	32.7 ± 0.4	20.1 ± 0.7	17.2 ± 0.9	71.6 ± 0.7	66.7 ± 0.8
0.16	32.3 ± 0.6	20.7 ± 1.0	17.2 ± 0.5	91.8 ± 0.5	72.6 ± 0.6
0.24	33.7 ± 0.5	19.7 ± 0.6	18.4 ± 0.6	84.7 ± 0.5	66.8 ± 1.0

Note: Pretreatment: 0.08–0.24 (g NH_3_)/(g biomass), S/L = 0.47, 24 h, 90 °C; Enzymatic hydrolysis: 15 (FPU (filter paper unit) CTec2)/(g glucan) loading, 50 °C, 150 rpm, 72 h.

**Table 3:** Effects of various parameters and their combinations on glucan and XMG digestibility (ANOVA).

Source	Enzymatic Digestibility [%]			
	Glucan		XMG	
	*F* Value	*p* Value	*F* Value	*p* Value
Temp	7.0205	0.0182	4.5518	0.0511
Time	1.1788	0.2947	0.1776	0.6799
NH_3_	1.2794	0.2758	0.9883	0.3370
S/L	1.0458	0.3227	0.1709	0.6855
Temp × Time	6.4939	0.0233	5.3117	0.0370
Temp × S/L	22.7188	0.0002	0.1994	0.6620
Temp × NH_3_	0.0025	0.9611	29.5164	<0.0001
Time × S/L	0.0466	0.8319	0.9617	0.3434
Time × NH_3_	0.7513	0.3997	18.9960	0.0007
S/L × NH_3_	3.1078	0.0983	1.03112	0.3271

Note: Pretreatment: 0.08, 0.16 and 0.24 (g NH_3_)/(g biomass), S/L = 0.29, 0.47, and 0.67, 24–120 h, 90–150 °C. Enzymatic hydrolysis: 15 (FPU CTec2)/(g glucan) loading, 50 °C, 150 rpm, 72 h. The probability level of 0.05 (*p* = 0.05) was used to test the significance.

**Table 4:** Effects of residual ammonia content on glucan digestibility under various reaction conditions.

Reaction Conditions	*R*^2^ Value for Glucan Digestibility		
	Low Severity	Medium Severity	High Severity
Time	0.0307	0.0298	0.0027
Temperature	0.0084	0.1352	0.0184
NH_3_ loading	0.0037	0.4113	0.2717
S/L	0.3950	0.1374	0.5607

Note: Low severity: Time = 24 h, temp. = 90 °C, NH_3_ loading = 0.08 (g NH_3_)/(g biomass), S/L = 0.29. Medium severity: time = 48–96 h, temp. = 120 °C, NH_3_ loading = 0.16 (g NH_3_)/(g biomass); S/L = 0.47. High severity: time = 120 h, temp. = 150 °C, NH_3_ loading = 0.24 (g NH_3_)/(g biomass), S/L = 0.67.

**Table 5:** A comparison of various ammonia pretreatment methods.

		ARP/LLARP	SAA	LMAA	LLAA
Catalysts		Aqueous NH_3_	Aqueous NH_3_	Gaseous NH_3_	Aqueous NH_3_
Reaction type		Flow-through	Batch	Semi-batch	Batch
Chemical loading		0.5 (g NH_3_)/(g biomass)	0.9 (g NH_3_)/(g biomass)	0.1 (g NH_3_)/(g biomass)	0.16 (g NH_3_)/(g biomass)
Water consumption ^1^		2.8 (g H_2_O)/(g biomass)	5.1 (g H_2_O)/(g biomass)	<1.0 (g H_2_O)/(g biomass)	<1.0 (g H_2_O)/(g biomass)
Temperature		170 °C	60 °C	90 °C	90 °C
Time		10 min	12 h	48 h	24 h
Pressure		2.5 MPa	-	-	-
Washing		Yes	Yes	No	No
Enzymatic digestibility ^2^	Glucan	92.5% (ARP) ^3^ 90.1% (LLARP)	85.3%	84.1%	91.8%
	XMG	78.0% (LLARP)	75.3%	73.6%	72.6%
Reference		[26,27]	[23]	[8,10]	This study

Note: ^1^ Water consumption does not include water for washing after pretreatment. ARP: ammonia recycle percolation, LLARP: low-liquid ammonia recycle percolation, SAA: soaking in aqueous ammonia, LMAA: low-moisture anhydrous ammonia, LLAA: low-liquid aqueous ammonia; ^2^ enzyme loading; 15 FPU/g-glucan, enzymatic digestibility after 72 h of hydrolysis; ^3^ enzyme loading; 10 FPU/g-glucan.
